# Sexually Transmitted Diseases—An Update and Overview of Current Research

**DOI:** 10.3390/diagnostics13091656

**Published:** 2023-05-08

**Authors:** Kristina Wihlfahrt, Veronika Günther, Werner Mendling, Anna Westermann, Damaris Willer, Georgios Gitas, Zino Ruchay, Nicolai Maass, Leila Allahqoli, Ibrahim Alkatout

**Affiliations:** 1 Department of Obstetrics and Gynecology, University Hospitals Schleswig-Holstein, Campus Kiel, Arnold-Heller-Strasse 3 (House C), 24105 Kiel, Germany; 2 German Center for Infections in Gynecology and Obstetrics, at Helios University Hospital Wuppertal, Heusnerstrasse 40, 42283 Wuppertal, Germany; 3 Department of Gynecology-Robotic Surgery at European Interbalkan Medical Center, 57001 Thessaloniki, Greece; 4 School of Public Health, Iran University of Medical Sciences (IUMS), Tehran 14167-53955, Iran

**Keywords:** sexually transmitted diseases, Chlamydia trachomatis, condyloma, pelvic inflammatory disease, infertility

## Abstract

A rise in the rates of sexually transmitted diseases, both worldwide and in Germany, has been observed especially among persons between the ages of 15 and 24 years. Since many infections are devoid of symptoms or cause few symptoms, the diseases are detected late, may spread unchecked, and be transmitted unwittingly. In the event of persistent infection, the effects depend on the pathogen in question. Manifestations vary widely, ranging from pelvic inflammatory disease, most often caused by Chlamydia trachomatis (in Germany nearly 30% of PID) or Neisseria gonorrhoeae (in Germany <2% of PID), to the development of genital warts or cervical dysplasia in cases of infection with the HP virus. Causal treatment does exist in most cases and should always be administered to the sexual partner(s) as well. An infection during pregnancy calls for an individual treatment approach, depending on the pathogen and the week of pregnancy.

## 1. Introduction

According to the WHO, more than a million sexually transmitted infections (STI) are diagnosed every day throughout the world [1]. On average, every year 374 million persons contract a new infection with one of four leading sexually transmitted pathogens: Chlamydia trachomatis, Trichomonas vaginalis, Neisseria gonorrhoeae, or human papillomaviruses [2].

In the USA and in Germany, an increasing number of infections have been observed especially among adolescents and young adults between the ages of 15 and 24 years [3].

The transmission routes of viruses, bacteria, fungi, or parasites in connection with sexual intercourse usually occurs through the exchange of infectious fluids or through direct skin contact (Table 1). However, many of these infections remain asymptomatic for a long period of time and are thus transmitted unwittingly or become persistent. This may cause long-term complications such as the pelvic inflammatory disease syndrome (PID), cervical dysplasia, or sterility. The gynecologist is then confronted with these conditions.

In the following, the diagnosis, symptoms, treatment, and prevention of a selection of sexually transmitted diseases are presented. In addition, a case report is included in this review to provide a more clinical context.

## 2. Brief Case History

**Figure 1:**
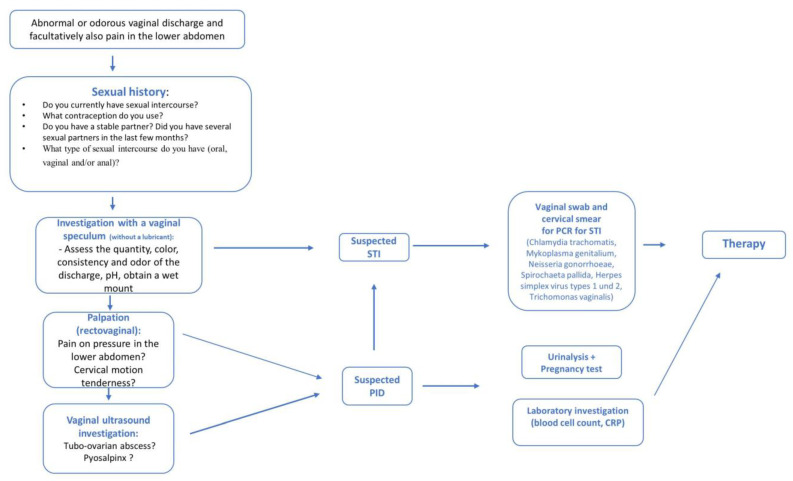
Flowchart of practical steps. Abbreviations: STI: Sexually transmitted infection, PID: Pelvic inflammatory disease; PCR: polymerase chain reaction.

A 29-year-old patient with increased vaginal discharge for two weeks presented at a gynecologist’s office. She reported pain in the lower abdomen, fever, and chills during the last two days. She had recently started a relationship with a man who had had a male partner previously. Her partner had been combating a bladder infection for a significant period of time. The investigation of the patient revealed an increased quantity of greenish vaginal discharge. Intracervical and intravaginal swabs were obtained with the speculum. Palpation of the vagina disclosed cervical motion tenderness and significant pain on pressure in the right-sided adnexa. The laboratory investigation revealed elevated leukocytes (17,000/µL) and C-reactive protein (CRP) levels (97mg/L). How would you proceed (Figure 1)?

## 3. Spectrum of Pathogens

Table 1 provides an overview of the spectrum of pathogens, divided into four groups: parasites, fungi, bacteria, and viruses. Furthermore, typical examples in each case are mentioned.

## 4. Etiology

Infection with a sexually transmitted disease usually occurs through the exchange of infectious fluids or by direct skin contact during sexual intercourse [1].

## 5. Predisposing Factors

The following risk factors apply to all sexually transmitted infections: frequent change of partners, young age, smoking, and drug abuse [7].

Furthermore, previous infections or a pre-existing infection may serve as predisposing factors for acquiring further pathogens [5]. For instance, a person infected with the human immunodeficiency virus (HIV) is exposed to a higher risk of infection, persistence of infection, the upward migration of other pathogens from the cervix or the vagina towards the uterus, and further upward migration of germs into the peritoneum [7]. A persistent HPV infection also favors additional vaginal and cervical infections [8]. In the vagina of premenopausal women, the presence of lactobacilli is a prerequisite for maintaining an acidic environment with a physiological pH between 4 und 4.4, which prevents the augmentation of pathogenic or potentially pathogenic germs [8]. Any disruption of this natural microbiome of the vaginal tract increases the risk of infection [8]. Thus, vaginal dysbiosis is a risk factor for infection with sexually transmitted pathogens and the emergence of a PID, with the subsequent complications of subfertility and ectopic pregnancy [9].

Note: Young women are especially at risk [4].

## 6. Prevention

The best preventive measure is to enlighten the population about the benefits of condoms [10]. The latter do not merely protect sexual partners from undesired pregnancy but also effectively hinder the spread of STIs [10]. Female patients should use a condom during vaginal as well as anal sex, if it is not used by the male partner [10].

Regardless of the physician’s specialty, he/she is advised to record the patient’s sexual history as part of the patient’s general medical history. In asymptomatic patients with a positive history, the physician could then initiate diagnostic investigations and, if needed, appropriate treatment on a timely basis [11].

In the event of treatment for a diagnosed STI, simultaneous treatment of the patient’s partner is mandatory [11]. Women must be urged to inform their last sexual partners and also be advised to abstain from sexual intercourse until the treatment has been concluded [12].

Hepatitis B and HPV can be prevented by vaccination. The latter should ideally be given prior to first sexual intercourse [13]. An increase in HPV vaccination rates would be very desirable [14].

Postexposure prophylaxis with 1 × 200 mg doxycycline also appears to reduce the risk for STIs. Both advantages and disadvantages are discussed with regard to this therapy. At present, however, the advantages outweigh the disadvantages, so the therapy is recommended [15].

## 7. Clinical Symptoms and Diagnostic Investigation

Infections may be accompanied by a variety of symptoms. However, many infections remain asymptomatic. Therefore, it is important to establish the risk of an STI on the basis of a patient’s medical history and then initiate timely treatment.

The following questions may be asked: Do you currently have sexual intercourse? If yes, do you have intercourse with a single partner or with changing sexual partners? How many partners have you had in the last six months? What contraception do you use? Do you use condoms? What type of sexual intercourse do you have (oral, vaginal, and/or anal)? Incorporating these few questions in the general medical history of the patient yields crucial additional information about the patient in just a few minutes (Table 2).

The investigation of sexually transmitted diseases should be aligned to the respective risk situation, sexual practices, and the existing symptoms. However, an inspection of sexual organs and the perianal region should be a basic element of the investigation. In women, the speculum should be used for inspection, for obtaining the pH value from the vaginal wall, and for obtaining a swab of the cervix for the PCR investigation and a wet mount (phase contrast × 400), followed by palpation. The pH value and the wet mount must be obtained before any contact with the ultrasound gel. Finally, depending on the symptoms, an additional vaginal ultrasound investigation may aid the diagnosis. This would be especially important in women with abdominal symptoms of long duration. Depending on individual sexual practices, the patient’s mouth and throat should be inspected, and swab specimens should be obtained if necessary [16].

Note: A comprehensive examination must include an inspection of the mouth and a throat swab.

### 7.1. Human Papillomaviruses

Human papillomaviruses are a type of small DNA virus that infects the squamous epithelium of the skin or the mucous membranes. The majority of infections are eliminated by the body’s immune system. However, if the viruses persist the patient may develop condylomas, precancerous conditions, or even invasive cancer over time [17,18].

More than 200 types of the HP virus have been classified so far. A distinction is made between high-risk and low-risk types with regard to their carcinogenic potential.

**Figure 2:**
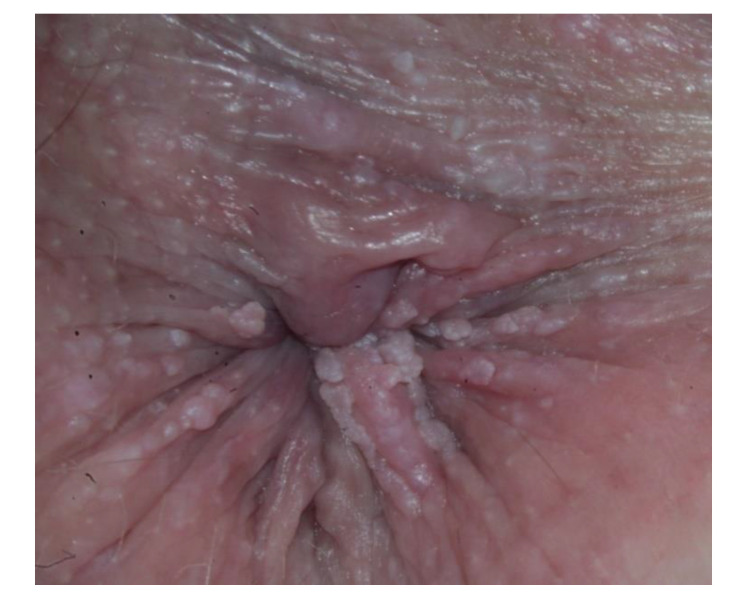
Perianal condylomata acuminata.

The low-risk HPV types 6 and 11 are found most commonly in anogenital condyloma acuminata (Figure 2) [19]. The incubation period is about two to three months [20]. The treatment of condyloma acuminata should be aligned to the location, number, and size of the warts. On the one hand, we have topical treatment, such as podophyllotoxin 0.5% solution, imiquimod 5% cream, or sinecatechins 10% ointment. A further alternative is surgery, which may consist of cryotherapy or the removal of the warts by means of electrocautery, laser, or scissors [21].

**Figure 3:**
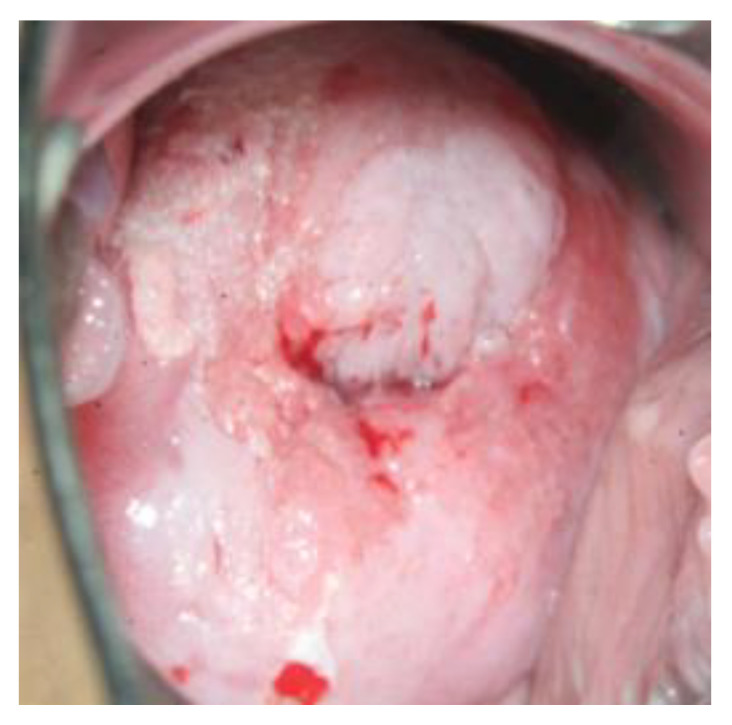
CIN III with major changes.

The high-risk HPV types 16 and 18 are responsible for 70% of HPV-induced cervical cancers and their preliminary stages or cervical intraepithelial neoplasia (CIN) [22]. The interval between an infection with an HP virus and the emergence of invasive cervical cancer is about 10 years [17]. After acetic acid staining, changes in the cervix on colposcopy are graded as minor or major (these may have a mosaic or dotted pattern) and should be investigated by performing a biopsy (Figure 3) [23]. This is usually done in the course of a so-called investigative colposcopy, which follows after an unusual PAP smear report and/or in case of a persistent HPV infection, as part of secondary prevention of cervical cancer [18].

Primary prophylaxis is provided by the previously mentioned vaccination against HP viruses in order to prevent an infection in the first place [17]. Since 2007, the vaccination is recommended twice for girls between the ages of 9 and 14 years, with an interval of 2–6 months between the two doses [17]. After the age of 14 years, the vaccination should be given three times in intervals of 2–6 months because the antibody response is weaker in older adolescents [17]. Since 2018, this recommendation also applies to boys [17]. Initially, there was a bivalent vaccine that only contained the high-risk types 16 and 18, but we now have a nonavalent vaccine that includes types 6, 11, 31, 33, 45, 52, and 58 [13].

Note: Girls and boys should be vaccinated between the ages of 9 and 14 years.

### 7.2. Chlamydia trachomatis (Serotypes D to K)

Chlamydia are the most common sexually transmitted pathogens and are mainly found in young women between the ages of 14 and 25 years [24].

A chlamydia infection is usually asymptomatic (70–90% of cases), which causes the pathogens to persist for as long as a few years [16]. Upward migration of the germs may trigger types of inflammation such as endomyometritis, urethritis, salpingitis, and PID, with the late sequelae of subfertility and infertility [24]. Chlamydia can be proven by the nucleic acid amplification test (NAAT), which can be performed on a first-void urine sample or an endocervical swab specimen. The latter is the method of choice because a higher concentration of pathogens is found in the cervix [16].

Once a chlamydia infection has been established, the patient as well as the patient’s sexual partner should be treated even in the absence of symptoms [25]. The partner should be treated by the general physician or the urologist. Sexual abstinence should be maintained until the treatment has been concluded and the success of treatment has been established (control investigation at the earliest after 4–6 weeks) in order to avoid a ping-pong effect [25]. The treatment may consist of a single dose of 1 g azithromycin or 100 mg doxocycline taken orally twice daily for seven to 10 days [16].

Caution: 70–90% of chlamydia infections remain subclinical [25].

Tip: The partner must also undergo treatment in order to avoid a ping-pong effect.

### 7.3. Mycoplasma genitalium

In the last few years, Mycoplasma genitalium has been recognized as a sexually transmitted pathogen responsible for genitourinary infections with a high risk of preterm birth [26]. Little is known about its prevalence among women in Germany because we lack comprehensive swab tests. However, among young men in the United Kingdom, it is found in 10–35% of patients with urethritis [26]. Mycoplasma genitalium is involved in cervicitis and PID in 20–25% of cases [26]. The symptoms are similar to those of Chlamydia trachomatis (discharge, urethritis (dysuria in 30%), cervicitis, salpingitis, and reactive arthritis), but 50% of these infections are also asymptomatic.

The swab is tested by the same method as that used for chlamydia, namely the nuclear acid amplification test (NAAT)/PCR [26].

### 7.4. Neisseria gonorrhoeae

Neisseria gonorrhoeae belongs to the group of Gram-negative diplococci. Gonococci possess fimbriae (pili) by which they can bind to the epithelial layer and are particularly adherent to the human urethral and cervical mucosa as well as the conjunctiva of the eyes [27]. Typical symptoms include dysuria, pollakisuria, and a purulent cervical or urethral discharge. The acute phase is followed by a chronic phase with much less suppuration. If the pathogens are spread in the body by the hematogenic route, the patient may develop monoarthritis or even gonococcal sepsis with pleuritis, meningitis, or endocarditis. Ascending infection may cause salpingitis or pelvic peritonitis and subsequent sterility or a high risk of ectopic pregnancy [12,27].

The recommended diagnostic procedure is to demonstrate the pathogen in cultures of urethral and cervical swab specimens of the gonococci. Gonorrhea can be evidenced very clearly during menstruation because the germs are especially prone to multiplication in a sanguineous milieu [27]. Due to increasing resistance, it is very important to test for resistance and initiate guideline-oriented antibiogram-based treatment in case first-line therapy fails [16]. Gonococci are highly sensitive and are able to survive in a culture transport medium for no longer than 2–4 h, especially in a warm environment. This problem does not exist in PCR testing, but the latter does not permit a resistance test [27].

The routine treatment is a single dose of 1–2 g ceftriaxone either by the intravenous or the intramuscular route. As co-infection with chlamydia is frequently present, patients with low compliance should also receive a single dose of 1 g azithromycin orally. In compliant patients, one may initially await the results of the swab test in order to address a proven co-infection in a targeted manner (Table 3) [16]. Here again, the partner must be treated simultaneously. Repeat swabs to check the success of treatment should—if subjected to a culture test—be obtained on the 5th and 10th day after the start of therapy. If both tests are negative, the patient may be considered cured [12].

### 7.5. Trichomonas vaginalis

A trichomonas infection is one of the most common sexually transmitted infections throughout the world, but its prevalence in Germany is low [28].

**Figure 4:**
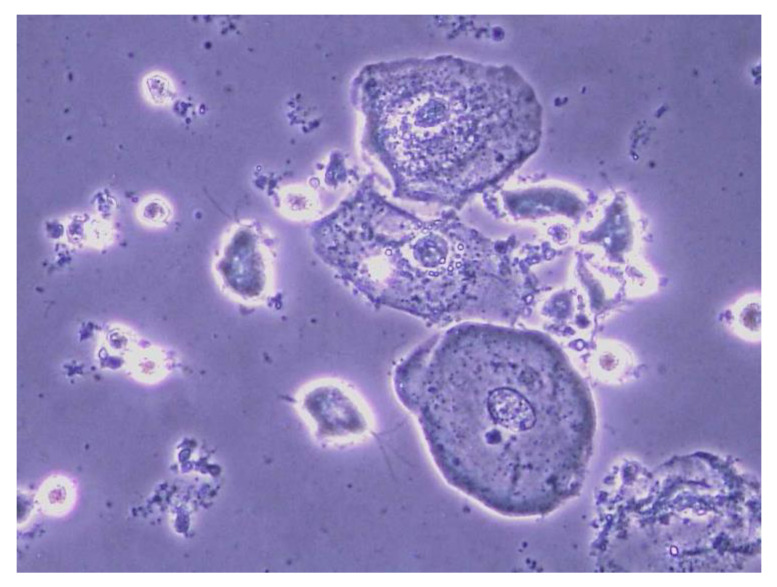
Wet mount of a patient with a Trichomonas vaginalis infection (phase contrast microscopy, 400-fold magnification, in 0.8% NaCl solution).

**Figure 5:**
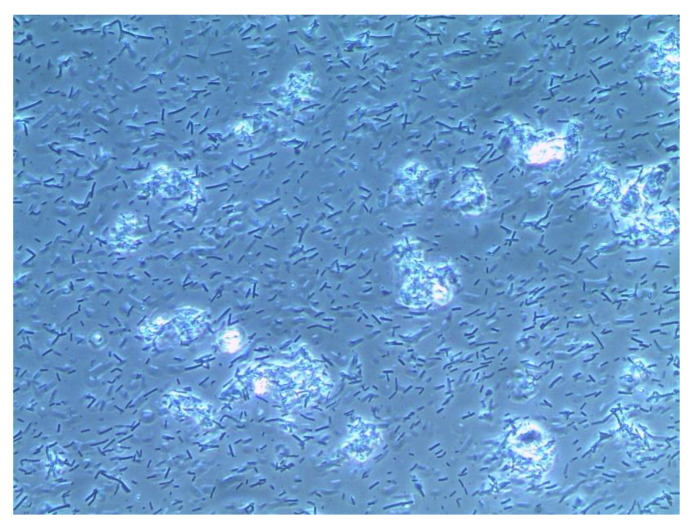
Premenstrual physiological wet mount (phase contrast microscopy, 400-fold magnification, in 0.9% NaCl solution): Figure 4 and Figure 5 were kindly provided by co-author Werner Mendling.

Trichomonads are protozoa that exist in different species as parasites in water, animals, and humans [28]. A trichomonas vaginalis infection initially causes a marked reproduction of protozoa in the vagina and a subsequent inflammatory reaction. The cervical glands release immunoglobulins which trigger the inflammatory reaction. As this reaction does not occur after hysterectomy, the trichomonads are able to grow unchecked. In the presence of bacterial vaginosis, the pH increases from 4.5 to 5.5. This is a “feel-good pH” for trichomonads, which is the reason why they occur more commonly in the presence of bacterial vaginosis [27]. Typical symptoms include a greenish-yellow discharge, itching, burning in the vestibulum, dyspareunia, dysuria, or cervix bleeding on contact. Clinically, however, the infection is asymptomatic in half of cases; this is equally true of men and women [27]. Trichomonads are identified directly on a wet mount of vaginal discharge. Under the microscope, one finds increased leukocytes, and trichomonads double the size of leukocytes. Trichomonads are pear-shaped, with a horned process at one end and four flagella at the other end (Figure 4) [28]. In comparison, Figure 5 shows a physiological wet mount with lactobacilli (Döderlein bacilli) and vaginal epithelial cells. The wet mount should be viewed soon after sampling because trichomonads do not survive cold temperatures or the incidence of light [28]. The sensitivity is strongly dependent on the investigator. Therefore, it would be advisable to obtain a swab from the vagina or urethra or a urine sample and demonstrate trichomonads by means of a nucleic acid amplification test (NAA) or a multiplex PCR or a biochemical bedside test [28].

Once the pathogen has been confirmed, the treatment of choice is 500 mg metronidazole, given orally twice daily for 7 days [16]. A control investigation should be performed no earlier than 3 weeks later. All sexual partners of the preceding 60 days should be treated simultaneously [29].

### 7.6. Herpes Simplex Virus (HSV), Types 1 and 2

A distinction is made between various herpes viruses (varicella-zoster virus, cytomegalovirus, HSV-1, and HSV-2). While HSV-1 (oral type) triggers labial herpes, according to the traditional view HSV-2 is responsible for genital symptoms [30]. According to reports from the USA, however, HSV-1 is now found more frequently in genital herpes infections than it was earlier. This is probably due to orogenital contact, which is particularly common among adolescents and young adults [31].

**Figure 6:**
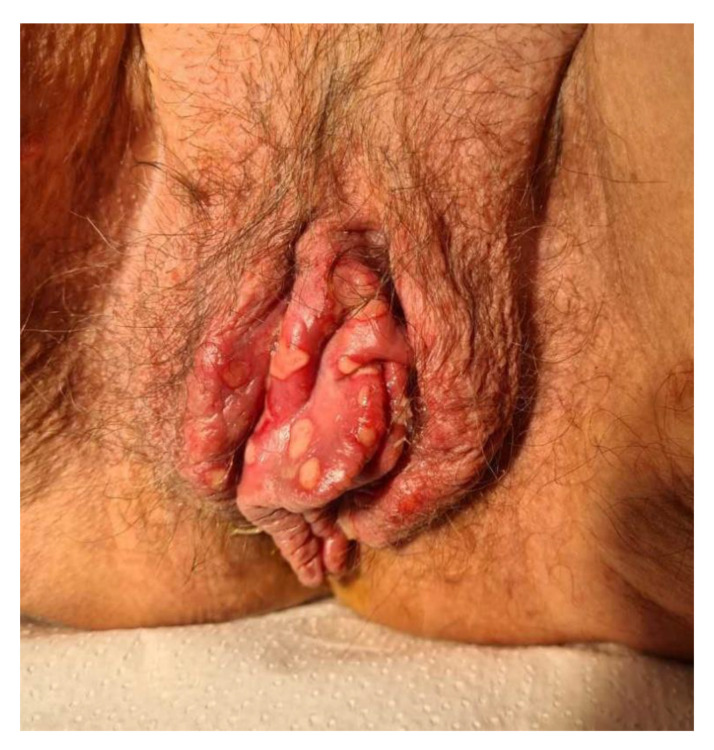
Genital herpes.

The viruses are transmitted by direct physical contact during sexual intercourse or orogenital intercourse. Transmission (viral shedding) may also occur without clinical symptoms. About a half of the infections are asymptomatic [32]. Typical clinical manifestations include an edema of the vulva with several small blisters on inflammatory skin after an incubation period of 3–8 days (Figure 6). The blisters erode over time and lead to painful ulcerations. Furthermore, the patient may experience general symptoms such as pain in the extremities or muscle pain, fever, or vomiting [32]. The typical clinical appearance is usually sufficient to establish the diagnosis. The recommended treatment is 200 mg aciclovir taken orally for 5 days [30]. Aciclovir ointment is used frequently but is considered ineffective and promotes (increasing) resistance to aciclovir [30].

### 7.7. Salpingitis, Pelvic Inflammatory Disease (PID)

PID is defined as an inflammation of the female pelvic organs with involvement of the peritoneum in the abdominal cavity, especially in the lower abdomen [7]. It usually occurs due to the ascension of pathogens, which may be transmitted during sexual intercourse. The infection spreads per continuitatem through the vagina/cervix, uterus, adnexa, and further into the abdominal cavity [7]. Cervicitis, endometritis, and salpingitis or adnexitis frequently cannot be differentiated from one another in terms of etiology, clinical appearance, or therapy [27]. This is because it is always an ascending disease from the cervix, with the exception of hematogenic genital tuberculosis which is rare in Germany, or the carried-over form of tuberculosis as in appendicitis. The term commonly used in the international published literature—pelvic inflammatory disease (PID)—expresses the fact that, in terms of pathomorphology, one may find accompanying inflammations such as parametritis, perimetritis, peritonitis, perihepatitis, perinephritis, perisplenitis, and tubo-ovarian abscess (TOA), as well as abscesses in the pouch of Douglas [33].

The most common sexually transmitted pathogens are Chlamydia trachomatis, Mycoplasma genitalium, and Neisseria gonorrhoeea [1]. In addition to the characteristic symptoms of the respective germs, the patient may develop general symptoms such as pain in the lower abdomen, fever, dyspareunia, and bleeding disorders [34].

**Figure 7:**
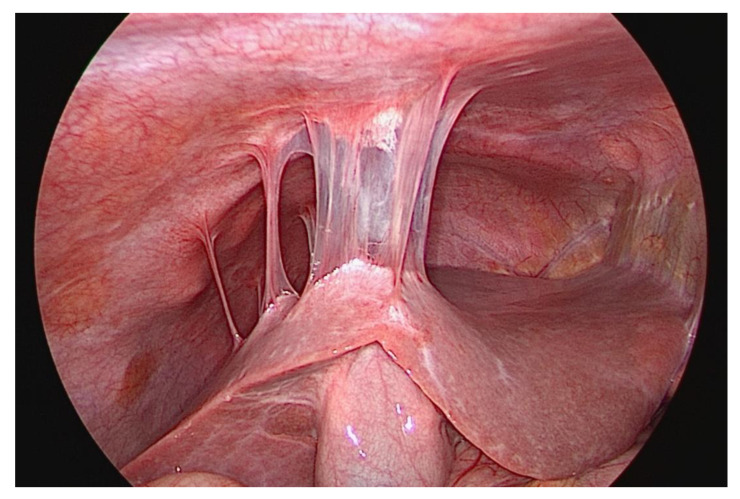
Fibrinous adhesions between the peritoneum and the liver surface (Fitz-Hugh–Curtis syndrome) [5].

PID may run an acute, subclinical, or chronic course (>30 days). The acute form is marked by severe pain in the lower abdomen, whereas the chronic type is marked by intermittent and less severe pain in the lower abdomen [7]. Right-sided pain in the upper abdomen may be indicative of a chronic PID or a Fitz-Hugh–Curtis syndrome (Figure 7). The latter is a form of perihepatitis with adhesions between the abdominal wall and the liver. Such adhesive bands in the peritoneum are usually discovered incidentally in a laparoscopy, several years after their emergence.

Predisposing factors for PID include smoking and lack of immunocompetence, such as an HIV infection. The most important risk factor, however, is an abnormal vaginal microbiome, especially in cases of bacterial vaginosis, and an STI. An intact vaginal microbiome, on the other hand, has a protective effect [33].

As in the case described earlier, the patients primarily experience pain in the lower abdomen. During the diagnostic investigation of PID, all other potential gynecological and non-gynecological differential diagnoses should be taken into account and ruled out (Table 4). A standard step is to obtain a urine sample and ensure the absence of a pregnancy.

The diagnosis of salpingitis/PID is unreliable and is established correctly by clinical investigation in a mere 60% of cases [33]. It is most reliably diagnosed by laparoscopy, which dates back to Jacobsen and Weström in Sweden as early as in 1969 [33]. In terms of method and the number of correctly diagnosed patients, this approach has not been superseded to date. One should obtain smears from the endings of the fimbriae (PCR test for Chlamydia trachomatis and Mycoplasma genitalium, PCR or culture including a general bacterial culture for gonococci). Fluid in the pouch of Douglas is not suitable for this purpose. The problem is that chlamydia, in the case of salpingitis, can be found at the fallopian tube in about 30% of cases but is seen in the cervix, urethra, or urine in just a half of these cases [35].

The diagnosis can be established with adequate certainty when the investigator finds a bacterial vaginosis or cervicitis either clinically or in the wet mount, in addition to pain in the lower abdomen and pressure-sensitive adnexa. If the patient also has fever in excess of 38.2 °C the diagnosis is accurate in about 80% of cases [34]. If one finds thickening of the adnexa and pathological inflammatory parameters in blood, the diagnosis is correct in more than 90% of cases [34]. A laparoscopy is considered unnecessary in this setting, especially in cases of mild disease [36].

In these instances, however, the clinician remains unaware of chlamydia at the fimbrial ends.

PID is treated with antibiotics. Mild disease may be treated on an outpatient basis with a combination of a single dose of 1–2 g ceftriaxone IV plus 100 mg doxycyclin given orally twice a day and 500 mg metronidazole given orally twice a day for 10 to 14 days [16]. In a case of more severe symptoms, the patient will need to undergo in-hospital treatment. The therapy initially consists of 2 g ceftriaxone given by the intravenous route once daily and 100 mg doxycyclin also given by the intravenous route twice daily for 3–5 days. This is followed by 100 mg doxycyclin taken orally twice a day and 500 mg metronidazole taken orally twice a day for a further 7–10 days [7].

Sexual abstinence is recommended until complete cure. Control swabs should be obtained from the patient and her sexual partner(s) at six weeks after conclusion of the antibiotic treatment [34].

## 8. Continuation of Case Report (Continued from Page 2)

**Figure 8:**
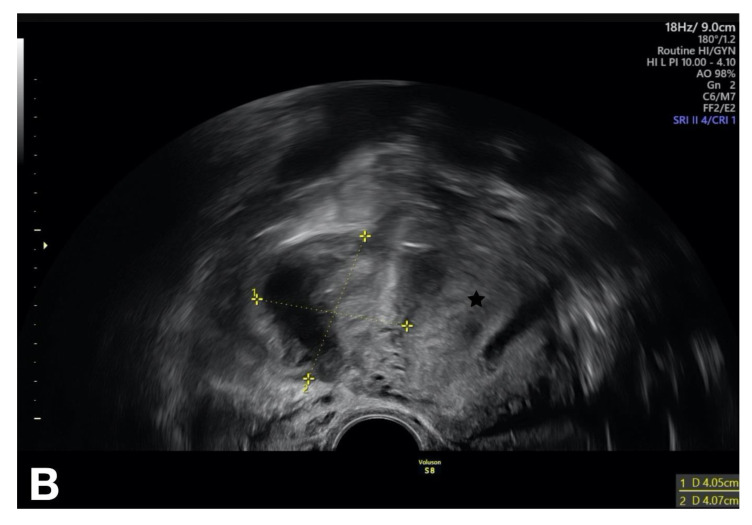
Vaginal ultrasound investigation showing the tubo-ovarian abscess in the right-sided adnexa. The uterus is marked with a star [5].

The patient reported above also had elevated inflammatory parameters. The vaginal ultrasound investigation, which was unpleasant for the patient, and the abdomen ultrasound investigation showed a thickened fallopian tube (>10 mm) on the right side. The Doppler investigation revealed increased vascularity. A hyperechogenic fluid was noted in the pouch of Douglas. We suspected a tubo-ovarian abscess on the right side as a complication of PID (Figure 8). We first initiated intravenous antibiotic therapy and then performed a secondary laparoscopy for drainage of the abscess after an interval of 3–4 days. Intraoperatively, we found a few adhesions; the right fallopian tube was adherent to the tubo-ovarian abscess. Therefore, we were unable to preserve the fallopian tube. In the region of the liver, we found adhesions to the abdominal wall by way of perihepatitis (Fitz-Hugh–Curtis syndrome). A surgical adhesiolysis is currently not recommended as a general measure because its value has not been clearly proven [36].

Table 5 summarizes the individual STIs.

## 9. Infertility

A further potential consequence of ascending infection, especially in the case of Chlamydia trachomatis and Neisseria gonorrhoeae, is infertility [4].

After a chlamydia infection and subsequent PID, the patient may develop a post-inflammatory occlusion of the fallopian tubes and intra-abdominal adhesions. One therefore finds higher rates of ectopic pregnancies or tubal factor infertility in these instances [37]. Furthermore, in some cases the fallopian tubes cannot be preserved after surgical treatment.

In Germany, all sexually active women below the age of 25 years are offered a chlamydia screening test at the expense of the health insurance in order to reduce the risk of infertility. However, it should be noted that in some cases, chlamydiae are only found in the funnel of the fimbriae and not in urine or in a swab specimen of the cervix [35].

## 10. Infections during Pregnancy

### 10.1. Screening during Pregnancy

Sexually transmitted diseases may occur during pregnancy. Depending on the time point and the respective pathogen, the infection may be associated with a number of risks. The maternity guidelines of the Federal Joint Committee recommend a serum screening for syphilis with the TPHA test, a serological test for antibodies to the rubella virus, and a test for genital Chlamydia trachomatis infection. The patient may be offered a pooled culture of samples taken from the rectum and vagina for Streptococcus agalactiae (B streptococci) for intrapartal prevention of neonatal early-onset sepsis, separate tests for HSV 1 and HSV 2 (as recommended in the guidelines) in case of suspected genital herpes, and screening for toxoplasmosis or a cytomegalovirus infection.

### 10.2. Chlamydia trachomatis

If a patient contracts an infection with Chlamydia trachomatis during pregnancy, she is subject to a higher risk of abortion, preterm birth, and a low birth weight [25]. In the presence of an active chlamydia infection during vaginal delivery, the germs are passed on to the newborn infant in 2/3rds of cases. In the event of an infection, the neonate may develop inclusion conjunctivitis (18–50%) or atypical pneumonia (11–18%) [25]. At the screening investigation, all pregnant women in Germany are tested for chlamydia at the start of their pregnancy in order to initiate prompt antibiotic treatment [38]. The maternal risk of postpartum endometritis is higher in the presence of a chlamydia infection. The symptoms include fever, mild pain at the margins of the uterus, and, in the case of anaerobic bacteria, additional fetid lochia, lochial congestion, and dysfunctional uterine bleeding typically 4–6 weeks after the delivery [25]. Especially in cases of repeat bleeding, the clinician should first rule out differential diagnoses such as retained placenta and then take a chlamydia infection into account [38]. If the patient develops endometritis, the clinician should take a swab to demonstrate the pathogen in question and then use an agent to promote contractions as well as antibiotic treatment with ampicillin [39].

### 10.3. Trichomonads

An infection with trichomonads during pregnancy raises the risk of preterm birth by a factor of 1.4 as well as the risk of premature rupture of the membranes. Therefore, treatment with metronidazole should be given during pregnancy [40].

### 10.4. Herpes Simplex Virus Types 1 and 2

Among mothers with an acute herpes simplex infection, only 5% of the fetuses are infected by the intauterine route, usually with HSV 2 [41]. Mothers with severe infection in the first trimester are subject to a high risk of abortion, stillbirth, or malformations in 50% of cases, and the risk of perinatal mortality is 50% [41].

In about 90% of cases, however, HSV (in about three fourths of cases it is HSV 2 that may cause a more severe course of disease) is transmitted during birth [41]. In a very small number of cases, the infection occurs during the first few hours or days after the delivery. In 50% of cases, the mother who passes on the infection to the infant has a primary HSV infection, whereas the transmission risk of an HSV recurrence during birth is just 2–5% [41]. However, it should be noted that in 70% of perinatal infections, the maternal HSV infection remains asymptomatic [41].

The child’s symptoms usually start 2 or 3 weeks after birth. Depending on the severity of disease, the infection is associated with a high lethality and a risk of permanent damage [41].

Therefore, the guidelines recommend that mothers with a primary infection be offered an elective Caesarean section; the risk of a perinatal herpes infection of the neonate is about 30% in these cases [41]. However, if a genital herpes infection is identified in the first or second trimester, one may anticipate maternal IgG antibodies with the ability to cross the placental barrier. In these cases, a vaginal delivery may be permitted in the presence of negative swab tests and no clinical symptoms [42]. Knowledge of the mother’s virus status helps in counseling the mother about the delivery. The father or any other infected person (midwife, obstetrician, nurse) may also infect the newborn.

## 11. Conclusions for Clinical Practice

1. In the presence of a genitourinary infection, the clinician must take sexually transmitted pathogens and simultaneous treatment of the partner into account.
2. The consequences of untreated STDs include, in addition to ascending infections, infertility and chronic recurrent lower abdominal pain.
3. Early vaccination for HP viruses at the age of 9–14 years and, as far as possible prior to first sexual intercourse, is associated with a markedly lower risk of the sequelae of HPV infection as well as the acquisition of other STI.
4. Young male and female patients should be informed early about the benefits of a condom in reducing and preventing the spread of STIs.
5. The preventive potential of a balanced vaginal microbiome is being recognized to an increasing extent through the options of modern non-culture-based techniques.

## Figures and Tables

**Table 1:** Spectrum of pathogens in STI [1,4,5,6].

Spectrum of Pathogens	
**Parasites**	Trichomonas vaginalis, Sarcoptes scabiei, Phthirus pubis
**Fungi**	Trichophyton mentagrophytes subspecies Note: Candida albicans is only facultatively pathogenic and does not cause STIs.
**Bacteria**	Chlamydia trachomatis (serotypes D-K, serotypes L_1_–L_3_), Klebsiella granulomatosis, Neisseria gonorrhoeae, Treponema pallidum, Haemophilus ducreyi; Mycoplasma genitalium
**Viruses**	Human papillomaviruses, Herpes simplex virus, HIV, Hepatitis B virus, Hepatitis C virus, Molluscum contagiosum virus, monkeypox virus

**Table 2:** Sexual history.

Information	Suggested Questions
Current sexual intercourse	Do you currently have sexual intercourse?
Number of sexual partners	Do you have a stable partner? Did you or did your sexual partner have several sexual partners in the last few months?
Sexual orientation	What gender do you feel attracted to?
Contraception	What contraception do you use? Do you take the pill? Do you use condoms? Do you have an intrauterine device?
Sexual practices	What type of sexual intercourse do you have (oral, vaginal and/or anal)?
Infections	Has an STD or an STI ever been diagnosed in your case? Do you currently have symptoms? Are you undergoing treatment at the present time?

**Table 3:** Antibiotic treatment of gonorrhea (modified according to [16]).

Therapy Options for Gonococcal Infections in Various Situations		
**Uncomplicated infection (urethra, cervix, rectum or pharynx)**	A: When the pathogen has not been established and the patient lacks compliance: ceftriaxone 1–2 g IV plus azithromycin 1 g, both given as single doses B: When the pathogen has not been established and the patient is compliant: ceftriaxone 1–2 g IV as a single dose C: In case of isolated detection of gonococci Single dose of ceftriaxone 1–2 g IV	Alternative: A single dose of ciprofloxacin 500 mg orally or: a single dose of ofloxacin 400 mg orally or: a single dose of azithromycin 2 g orally
**Gonococcal infection during pregnancy or lactation**	Ceftriaxone 1 g IV	Alternative in case of proven sensitivity: single oral dose of 2 g azithromycin

**Table 4:** Differential diagnosis of PID (modified according to [33,34].

	Acute Pain in the Lower Abdomen	Chronic Pain in the Lower Abdomen
Gynecology:	Torsion of the adnexa	Endometriosis
	Ectopic pregnancy	Myoma
	Ovarian cyst rupture	Allen–Masters syndrome
Other specialties:	Acute appendicitis	Sigmoid diverticulitis
	Acute cystitis	Psychosomatic
	Others	Chronic appendicitis
		Adhesions

**Table 5:** Summary of STIs (modified according to [1,5,16].

STI	Pathogen	Latency	Symptoms	Treatment	Complication
Genitourinary Chlamydia infection	Gram-negative bacteria		70–90% asymptomatic, purulent cervical/urethral discharge, endometritis, salpingitis, perihepatitis	Azithromycin, doxycyclin	Reactive arthritis, Fitz-Hugh–Curtis syndrome, infertility, Reiter’s syndrome, neonate: conjunctivitis,
Genital herpes	DNA viruses	3–8 days (may even take several months or years)	Genital edema and blisters	Aciclovir	Recurrent infections, sepsis, encephalitis, neonatal sepsis,
HPV infections	DNA viruses	Months or years	Condylomata acuminata in the genitourinary tract	Laser treatment, surgical removal, imiquimod	Cervical cancer
Trichomoniasis	Protozoa		Burning, itching, yellowish-green discharge	Metronidazole	
Gonorrhea	Gram-negative diplococci Neisseria gonorrhoeae	2–7 days	Purulent vaginal discharge, dysuria, pollakisuria	Ceftriaxone + azithromycin, cefixime	PID, epidydimitis, sepsis, septic arthritis, neonatal conjunctivitis

## Data Availability

The datasets analyzed for the current study are available from the corresponding author on reasonable request.

## Abbreviations

PID	Pelvic inflammatory disease
STI	Sexually transmitted infection
HPV	Human papillomaviruses
CRP	C-reactive protein
PCR HIV	Polymerase chain reaction Human immunodeficiency virus
STD	Sexually transmitted diseases
GV	Sexual intercourse
CIN	Cervical intraepithelial neoplasia
NAAT	Nucleic acid amplification test
HSV	Herpes simplex virus
TOA	Tubo-ovarian abscess
